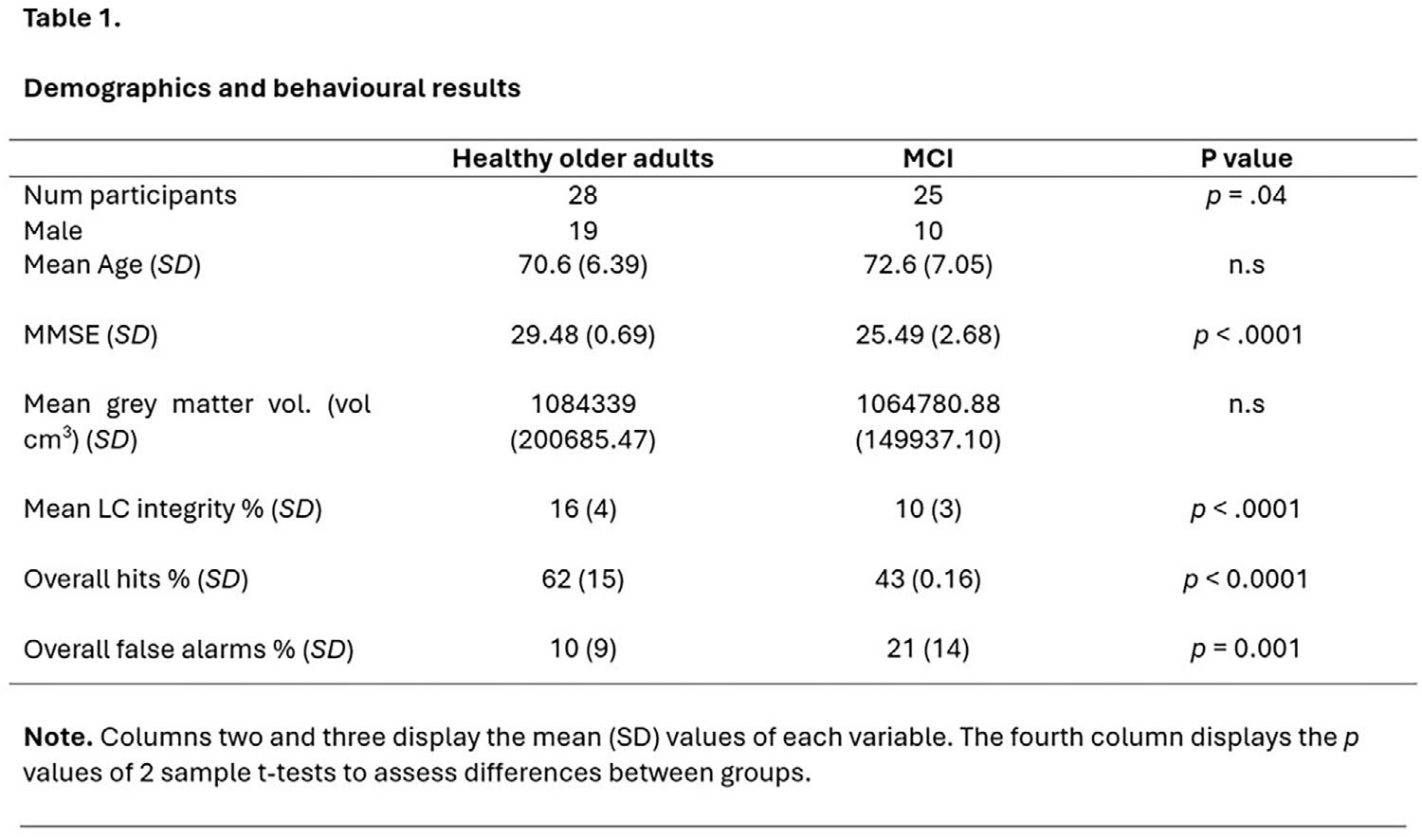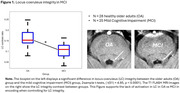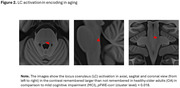# Brainstem and midbrain structures involved in memory encoding in healthy aging and mild cognitive impairment, a 3T task‐fMRI study

**DOI:** 10.1002/alz.095189

**Published:** 2025-01-09

**Authors:** Lucía Penalba‐Sánchez, Yeo‐Jin Yi, Elif Kurt, Grazia Daniela Femminella, Clare Loane, Millie Duckett, Martina F Callaghan, Nikolaus Weiskopf, Ray Dolan, Robert J Howard, Emrah Düzel, Dorothea Hämmerer

**Affiliations:** ^1^ Institute of Cognitive Neurology and Dementia Research (IKND), Otto‐von‐Guericke University Magdeburg, Magdeburg Germany; ^2^ German Center for Neurodegenerative Diseases (DZNE), Magdeburg Germany; ^3^ Aziz Sancar Institute of Experimental Medicine, Istanbul University, Istanbul, Istanbul Turkey; ^4^ University of Naples Federico II, Napoli Italy; ^5^ Institute of Cognitive Neuroscience, University College London (UCL), London United Kingdom; ^6^ Wellcome Centre for Human Neuroimaging, University College London (UCL), Queen Square Institute of Neurology, London United Kingdom; ^7^ Max Planck Institute for Human Cognitive and Brain Sciences, Leipzig Germany; ^8^ Institute of Neurology, University College London (UCL), London United Kingdom; ^9^ Felix Bloch Institute for Solid State Physics, Faculty of Physics and Earth Sciences, Leipzig University, Leipzig Germany; ^10^ Max Planck Centre for Computational Psychiatry and Ageing, University College London, London, London United Kingdom; ^11^ Division of Psychiatry, University College London, London United Kingdom; ^12^ Center for Behavioral Brain Sciences (CBBS), Magdeburg Germany; ^13^ University Hospital Magdeburg, Magdeburg Germany; ^14^ Institute of Cognitive Neurology and Dementia Research (IKND), Otto‐von‐Guericke University, Magdeburg Germany; ^15^ Department of Neurology, Otto‐von‐Guericke University, Magdeburg Germany; ^16^ Department of Psychiatry and Psychotherapy, Otto‐von‐Guericke University, Magdeburg Germany; ^17^ University of Innsbruck, Innsbruck Austria

## Abstract

**Background:**

Memory decline, which is especially prevalent in Alzheimer’s disease (AD), has been studied via fMRI, primarily focusing on the prefrontal cortex and hippocampus. However, emerging evidence suggests that the brainstem, alongside various midbrain regions, is an initial target for pathological processes like hyperphosphorylated TAU protein accumulation. Among these, the locus coeruleus, a noradrenergic nucleus in the pons, projects to critical midbrain areas supporting memory encoding. Hence, our study aimed to investigate BOLD task activations in AD relevant to memory, while focusing on differences in responses to emotional versus neutral stimuli in the brainstem and midbrain.

**Method:**

Using event‐related fMRI, 53 subjects (28 healthy older adults, 25 with mild cognitive impairment (MCI)) (see table 1) underwent an incidental recognition memory task involving emotional and neutral images. Memory tests followed immediately, and 4 hours after encoding. Group differences in brain activations for remembered versus not remembered images using the study template were examined.

**Result:**

Results revealed a trend for greater activation in the left caudate nucleus in older adults, compared to those with MCI, when subsequently remembered items were compared with not remembered ones (small volume correction (SVC), cluster level *p*FWE‐corr = 0.08). Similarly, a significant increased activation was observed in the locus coeruleus (SVC, cluster level *p*FWE‐corr = 0.018). However, after adjusting for group and individual differences in LC integrity and global grey matter volume (GMV), no significant differences persisted, suggesting that structural changes contribute significantly to differences in LC activation between healthy controls and MCI participants (see Figures 1 and 2).

**Conclusion:**

In conclusion, our findings underscore the caudate nucleus’s role in memory encoding for healthy older adults versus those with MCI. A decline in LC function in MCI appears related to a decline in LC integrity. These insights contribute to understanding memory mechanisms in healthy aging versus MCI. Future studies are needed to explore potential neural memory compensatory processes in MCI.